# Identification of cell-surface markers for detecting breast cancer cells in ovarian tissue

**DOI:** 10.1007/s00404-016-4036-7

**Published:** 2016-03-05

**Authors:** Inge T. A. Peters, Carina G. J. M. Hilders, Cornelis F. M. Sier, Alexander L. Vahrmeijer, Vincent T. H. B. M. Smit, J. Baptist Trimbos, Peter J. K. Kuppen

**Affiliations:** Department of Gynecology, Leiden University Medical Center, Leiden, The Netherlands; Department of Gynecology, Reinier de Graaf Hospital, Delft, The Netherlands; Department of Surgery, Leiden University Medical Center, Leiden, The Netherlands; Department of Pathology, Leiden University Medical Center, Leiden, The Netherlands

**Keywords:** Autotransplantation, Ovarian tissue, Breast cancer, Tumor markers, Fertility preservation

## Abstract

**Purpose:**

The safety of ovarian tissue autotransplantation in oncology patients cannot be ensured, as current tumor-detection methods compromise the ovarian tissue viability. Although non-destructive methods (for instance near-infrared fluorescence imaging) can discriminate malignant from healthy tissues while leaving the examined tissues unaffected, they require specific cell-surface tumor markers. We determined which tumor markers are suitable targets for tumor-specific imaging to exclude the presence of breast cancer cells in ovarian tissue.

**Methods:**

Immunohistochemistry was performed on formalin-fixed, paraffin-embedded specimens of ten ovaries from premenopausal patients. Additionally, we screened a tissue microarray containing tumor tissue cores from 24 breast cancer patients being eligible for ovarian tissue cryopreservation. The following cell-surface tumor markers were tested: E-cadherin, EMA (epithelial membrane antigen), Her2/neu (human epidermal growth factor receptor type 2), αvβ6 integrin, EpCAM (epithelial cell adhesion molecule), CEA (carcinoembryonic antigen), FR-α (folate receptor-alpha), and uPAR (urokinase-type plasminogen activator receptor). For each tumor, the percentage of positive breast tumor cells was measured.

**Results:**

None of the ten ovaries were positive for any of the markers tested. However, all markers (except CEA and uPAR) were present on epithelial cells of inclusion cysts. E-cadherin was present in the majority of breast tumors: ≥90 % of tumor cells were positive for E-cadherin in 17 out of 24 tumors, and 100 % of tumor cells were positive in 5 out of 24 tumors.

**Conclusions:**

Of the markers tested, E-cadherin is the most suitable marker for a tumor-specific probe in ovarian tissue. Methods are required to distinguish inclusion cysts from breast tumor cells.

## Introduction

Premature ovarian failure is the most common long-term major adverse effect in premenopausal women following chemotherapy [1]. Because loss of fertility can significantly decrease quality of life [2], considerable effort has been devoted to offer these patients options for preserving their fertility. These options currently include cryopreservation of embryos and/or oocytes. Besides, autotransplantation of pretreatment cryopreserved ovarian tissue is becoming more prevalent and is considered predominantly feasible for both prepubescent girls and women who cannot postpone adjuvant therapy [3–5].

Although autotransplantation of frozen-thawed ovarian tissue has improved greatly in recent years [6, 7], its safety is questionable for certain types of cancer at risk of ovarian involvement, as it remains uncertain whether the transplanted cortical ovarian strips contain metastatic cells. This uncertainty arises from the highly damaging effects of currently available tumor-detection methods (e.g., PCR, immunohistochemistry) on tissue viability [8, 9]. Therefore, traditional screening is performed using a limited number of ovarian strips that are ultimately not transplanted. As a consequence of this approach, autotransplanting ovarian tissue involves the risk of reimplanting diseased cells that can lead to cancer relapse in some patients.

To safeguard the transfer of cortical ovarian tissue to the patient, methods must be developed in which tumor cells can be detected in ovarian autografts while preserving the tissue’s reproductive function. Near-infrared fluorescence (NIRF) imaging might be a suitable approach, as this technique can safely distinguish malignant tissues from non-malignant tissues in real time while leaving the tissues viable [10]. A NIRF probe consists of a fluorophore that emits light in the near-infrared spectrum (*λ* = 700–900 nm) conjugated to an antibody or peptide with high affinity for a protein marker expressed selectively at the cell surface of tumor cells [11, 12].

The first step towards developing tumor-specific imaging is the identification of protein markers that are present selectively at the cell surface of tumor cells, but absent on cells within the normal ovarian cortex. Because breast cancer is one of the primary indications for cryopreservation of ovarian tissue [13–16] and breast cancer metastases in the ovaries have been reported with a prevalence ranging from 13 to 47 % [8, 17], we examined a panel of cell-surface markers known to be expressed by breast cancer cells. This panel included human epidermal growth factor receptor type 2 (Her2/neu) [18, 19], E-cadherin [20], and carcinoembryonic antigen (CEA) [21]. In addition, we tested several markers involved in tumor invasion and migration, including epithelial cell adhesion molecule (EpCAM) [22, 23], αvβ6 integrin [24], urokinase-type plasminogen activator receptor (uPAR) [25, 26], and epithelial membrane antigen (EMA, also known as MUC1) [27, 28]. Lastly, we included folate receptor-alpha (FR-α), which is expressed in several tumor types but not in normal ovarian tissue [29]. We excluded cytokeratin CAM 5.2, gross cystic disease fluid protein-15 (GCDFP15), Wilms’ tumor antigen-1 (WT1), mammaglobin 1, and cytokeratin 7 (CK-7), which were used previously by Sánchez-Serrano et al. [30] and Rosendahl et al. [31], as these proteins are not expressed at the cell surface and therefore not suitable as a target for tumor-specific imaging.

In this study, we measured the expression levels of the above-mentioned markers in breast cancer cells obtained from patients who were potentially eligible for cryopreservation of ovarian tissue. In addition, we compared these expression levels to expression in normal ovarian tissues.

## Materials and methods

### Tissue specimens

#### Control ovaries

Formalin-fixed, paraffin-embedded (FFPE) specimens of control ovaries obtained from premenopausal patients who underwent a unilateral or bilateral oophorectomy in 2001–2012 were selected from the archives of the Department of Pathology at the Leiden University Medical Center (LUMC). The clinical data were extracted from the patients’ medical records. Indications for surgery included suspected malignancy in the contralateral ovary, early-stage uterine sarcoma, endometrial carcinoma, squamous cell carcinoma of the cervix, or enlarged ovary during pregnancy. *BRCA* mutation carriers and women with unknown *BRCA* mutation status were excluded. Patients who used a gonadotropin-releasing hormone (GnRH) agonist or oral contraceptives prior to oophorectomy were excluded to ensure that only functionally active ovaries were studied. A pathologist specialized in gynecology confirmed the absence of overt abnormalities in the ovaries by reviewing hematoxylin-and-eosin-stained sections. A total of ten control ovaries from ten different patients were included.

#### Breast cancer tissue

Breast tumor samples were collected from 24 patients who were potentially eligible for cryopreservation of their ovarian tissue based on the inclusion criteria established by the Dutch Network of Fertility Preservation [32]. All women were ≤35 years of age and were diagnosed with invasive breast carcinoma for which they were treated surgically at the LUMC in 1997–2009. The following data were obtained from the medical records: age at the time the tissue was obtained, TNM (tumor/node/metastasis) stage, histological subtype, Scarff-Bloom-Richardson (SBR) grade, and expression of the estrogen and progesterone receptors. All patients were eligible for adjuvant chemotherapy based on the current protocols, and none was diagnosed with distant metastases.

### Immunohistochemistry

Immunohistochemistry was performed on 4-μm thick FFPE sections of control ovaries and 4-μm thick slices of a tissue microarray (TMA) containing invasive breast tumor cores. To generate the TMA, tissue biopsies measuring 1.0 mm in diameter were taken in triplicate from representative regions of the FFPE tumor samples and arrayed into a new recipient paraffin block using TMA Master (3DHistech, Hungary). The tissue sections were deparaffinized in xylene, rehydrated in a stepwise series of graded alcohol solutions, and rinsed in distilled water. After blocking endogenous peroxidase activity with 0.3 % hydrogen peroxide for 20 min, heat-induced antigen retrieval was performed by placing the slides in EnVision Flex Target Retrieval Solution high pH/low pH in PT Link (Dako, Denmark). EpCAM and αvβ6 integrin epitopes were unmasked by 30-min incubation with 0.125 % trypsin and 0.4 % pepsin, respectively, at 37 °C. The sections were incubated overnight in a humidified chamber at room temperature with primary antibodies against Her2/neu (ERBB2, rabbit polyclonal, Dako), E-cadherin (NCH38, mouse monoclonal, Dako), EpCAM (323/A3, mouse monoclonal, provided by the Department of Pathology, LUMC, the Netherlands), CEA (A0115, rabbit polyclonal, Dako), αvβ6 integrin (6.2A1, mouse monoclonal, Cell Essentials), uPAR (ATN615, mouse monoclonal, kindly provided by Prof. A.P. Mazar, Northwestern University, Evanston, IL), or EMA (E29, mouse monoclonal, Dako); all primary antibodies were used at their predetermined optimal dilution. Some sections were incubated with an antibody against FR-α (26B3.F2, mouse monoclonal, Biocare Medical) for 60 min in accordance with the manufacturer’s instructions. After incubation with the primary antibody, the sections were rinsed with PBS, incubated with secondary antibody (anti-mouse or anti-rabbit EnVision; Dako) for 30 min, and visualized using liquid DAB+substrate buffer (Dako). The sections were counterstained with Mayer’s hematoxylin solution, dehydrated, and permanently mounted with Pertex (Leica Microsystems, Germany). For each immunostain, a positive control expressing the antigen of interest was included. The primary antibody was omitted as a negative control.

### Image capture and quantification of immunoreactivity

The immunostained slides were scanned using a Pannoramic MIDI digital slide scanner (3DHistech, Hungary). Immunohistochemical staining of the ovary sections was evaluated by the primary researcher (I.P.) and an experienced pathologist specialized in gynecology (V.S.). In each breast tumor tissue core sample, the percentage of breast tumor cells and the percentage of positively stained membranes among the malignant cells were scored by two independent observers (I.P. and R.V.). In the event of a major discrepancy, the observers reached consensus regarding a final score. The tumor cell membranes were considered positive if they showed immunoreactivity of any intensity. A weighted scoring method based on the size of the tumor area in each tumor core was used to calculate the percentage of positive membrane-stained tumor cells in each sample.

### Statistical analysis

Statistical analysis was performed using SPSS version 20.0 (IBM, Armonk, NY). Inter-observer agreement was calculated using the Pearson correlation coefficient. The suitability threshold for the putative NIRF probe targets was set at 80, 90, or 100 % of tumor cells expressing the antigens.

## Results

### Control ovaries

**Fig. 1:**
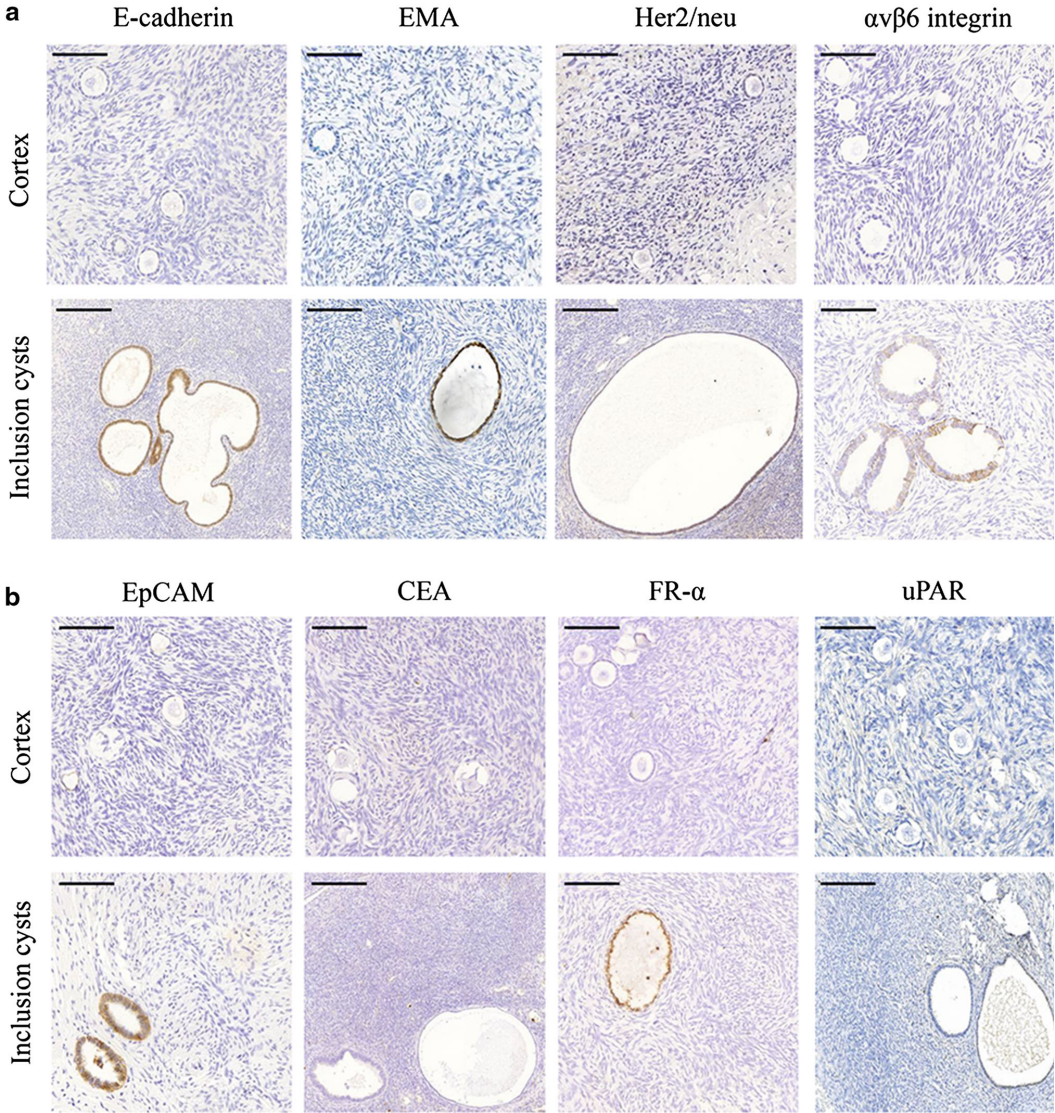
**a** Immunohistochemical expression of E-cadherin, EMA, Her2/neu and αvβ6 integrin in ovarian cortices and inclusion cysts. Stromal cells stained negative, but E-cadherin, EMA, Her2/neu and αvβ6 integrin showed expression at the epithelial cells of inclusion cysts. *Scale bars* in the *upper panel* represent 100 μm and *scale bars* in the *lower panel* represent 200 μm. **b** Immunohistochemical expression of EpCAM, CEA, FR-α and uPAR in ovarian cortices and inclusion cysts. Stromal cells stained negative, but EpCAM and FR-α showed expression at the epithelial cells of inclusion cysts. *Scale bars* in the *upper panel* represent 100 μm and *scale bars* in the *lower panel* represent 200 μm

**Fig. 2:**
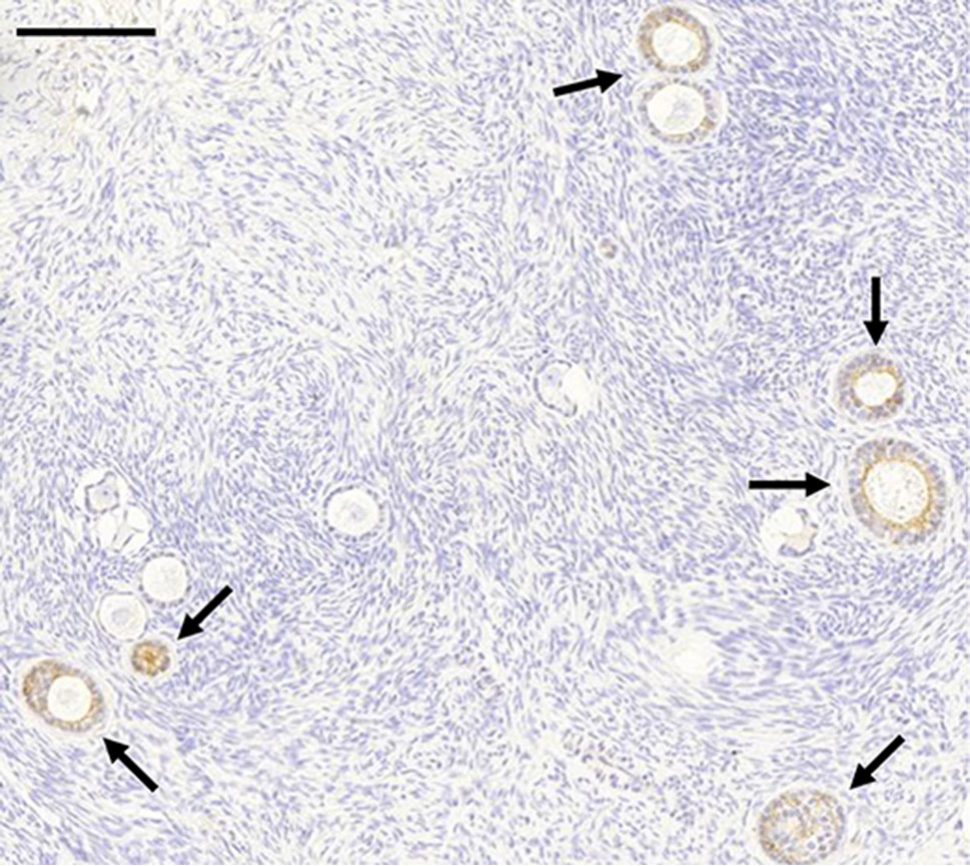
Immunohistochemical staining of E-cadherin showed moderate expression in the granulosa cells of primary follicles in the ovarian cortex (*arrows*). *Scale bar* represents 200 μm

A histological analysis showed that all ovaries contained follicles. The cortex of each ovary was negative for immunohistochemical staining by all markers tested. In contrast, all markers (except CEA and uPAR) were detected at the plasma membrane of epithelial cells in inclusion cysts (Fig. 1a, b). These inclusion cysts were present in five of the ten ovaries. In addition, E-cadherin was expressed at moderate levels in the granulosa cells of primary follicles (Fig. 2).

### Breast cancer tissue

The median age at the time of diagnosis was 32 years (range 21–35 years) for the 24 patients included in the TMA analysis. Twenty-three patients were diagnosed with ductal breast cancer, and the remaining patient was diagnosed with lobular breast carcinoma. The characteristics of these 24 patients and their tumors are summarized in Table 1.

**Table 1:** Clinicopathologic characteristics in premenopausal patients with primary invasive breast cancer

Characteristic	*N* = 24
Age at diagnosis, years—median (range)	32.0 (21–35)
Tumor size, mm—median (range)	20.5 (10–45)
Tumor stage, no. (%)	
pT1	11 (45.8)
pT2	12 (50.0)
pT3	1 (4.2)
pT4	0 (0.0)
Lymph node involvement, no. (%)	
pN0	13 (54.2)
pN1	11 (45.8)
Scarff-Bloom-Richardson grade, no. (%)	
I	2 (8.3)
II	9 (37.5)
III	13 (54.2)
Histological subtype, no. (%)	
Ductal	23 (95.8)
Lobular	1 (4.2)
Estrogen receptor, no. (%)	
Negative	12 (50.0)
Positive	9 (37.5)
Unknown	3 (12.5)
Progesterone receptor, no. (%)	
Negative	15 (62.5)
Positive	6 (25.0)
Unknown	3 (12.5)

#### Expression of investigated markers

**Fig. 3:**
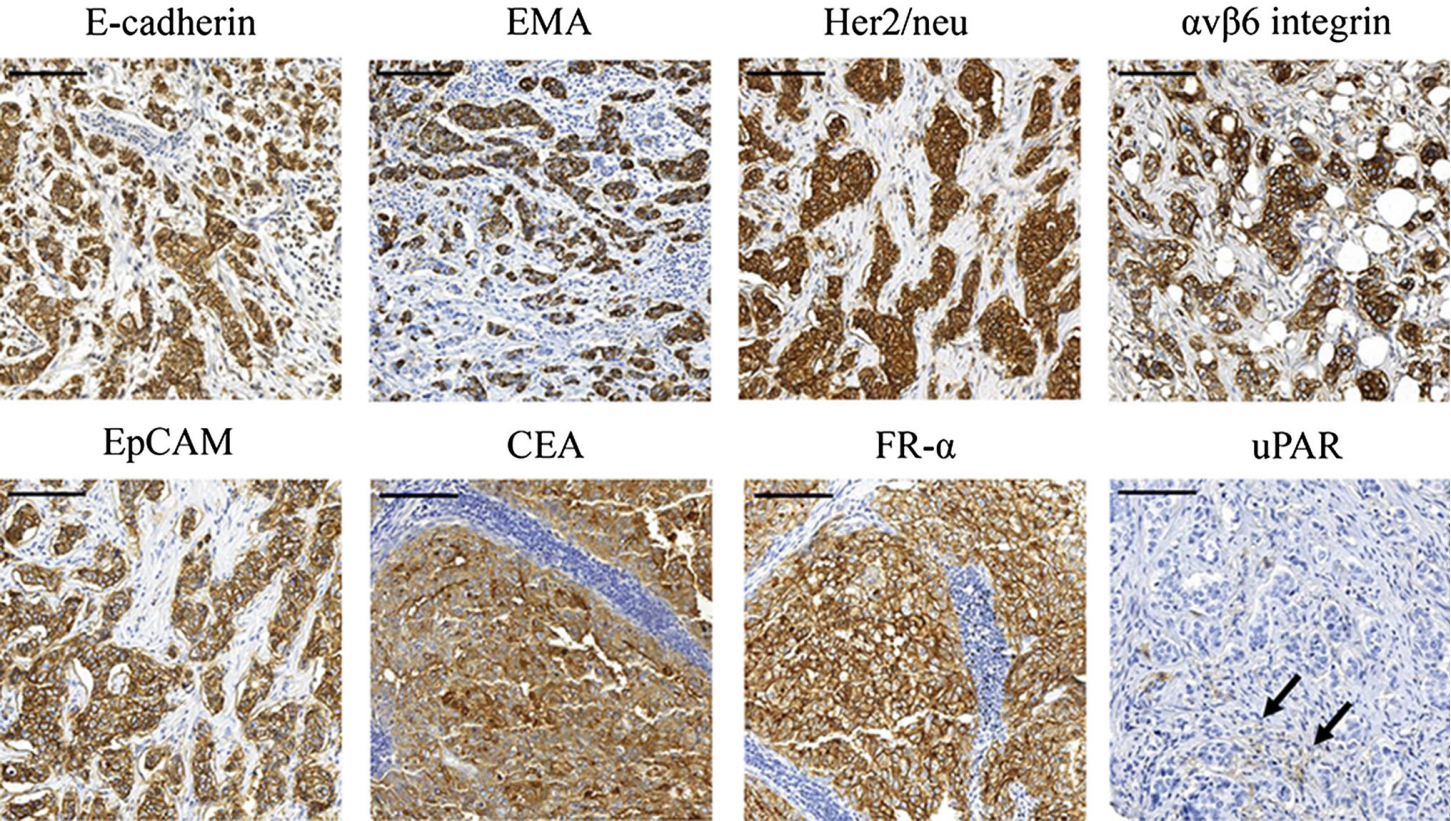
Immunohistochemical expression of E-cadherin, EMA, Her2/neu, αvβ6 integrin, EpCAM, CEA, FR-α and uPAR in invasive breast cancer. uPAR was barely expressed in stromal cells surrounding the tumor (*arrows*). *Scale bars* represent 100 μm

Microscopic quantification of marker levels was possible in all breast tumor samples. Strong correlation was obtained between the scoring results obtained by the two observers; the median *R*^2^ was 0.746 (range 0.626–0.818). E-cadherin, EMA, Her2/neu, CEA, and uPAR staining was positive in both the plasma membrane and cytoplasm of the breast cancer cells, whereas αvβ6 integrin, EpCAM, and FR-α staining was confined to the membrane. In addition, uPAR staining was observed in stromal cells surrounding the tumor cells (Fig. 3).

The median (range) percentage of positive tumor cells was 94 % (5–100) for E-cadherin, 78 % (13–100) for EMA, 61 % (11–100) for Her2/neu, 56 % (2–100) for αvβ6 integrin, 54 % (0–100) for EpCAM, 23 % (0–100) for CEA, and 3 % (0–100) for FR-α. uPAR was expressed in extremely few tumor and stromal cells, 0 % (0–11) and 0 % (0–14), respectively (Table 2).

**Table 2:**
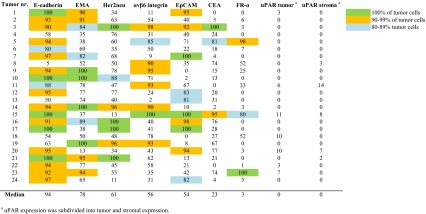
The percentage of tumor cells in each tumor showing positive expression for the investigated tumor markers

#### Potential targets for imaging

Given that breast cancer is relatively heterogeneous and that the expression of antigens varied among the tumors examined (Table 2), targeting one membrane protein would likely be insufficient for detecting all possible tumor cells in each patient. Therefore, to facilitate the selection of possible targets, we used suitability thresholds set at 80, 90, and 100 %, corresponding to the percentage of tumor cells that expressed the various antigens.

**Fig. 4:**
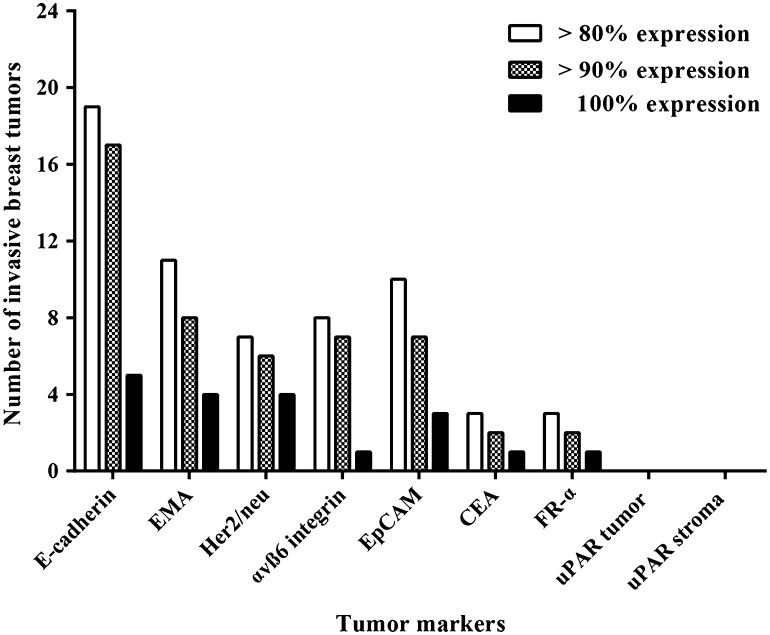
Suitability of tumor markers to use as a target for the detection of tumor cells in premenopausal women with invasive breast cancer (*n* = 24). *Columns* represent the number of tumors in which at least 80, at least 90 or 100 % of the tumor cells showed expression of the tumor markers. For uPAR, stromal cell expression is also shown

Figure 4 summarizes the suitability of each tumor marker for detecting invasive tumor cells in the 24 patients who were diagnosed with breast cancer. Based on this analysis, E-cadherin was identified as the most suitable marker for detecting breast cancer cells; specifically, E-cadherin was present in 100 % of cells in five tumors, and this marker was present in ≥90 % of cells in 17 tumors. The seven tumors with <90 % positivity for E-cadherin were positive for the markers EMA (1 tumor; 100 % of cells detected), αvβ6 integrin (3 tumors; 78–93 % of cells detected), EpCAM (1 tumor; 81 % of cells detected), E-cadherin (1 tumor; 80 % of cells detected), and Her2neu (1 tumor; 76 % of cells detected). Two tumors had <80 % positivity for all of the markers tested (Table 2). In these two tumors, 76 and 78 % of the tumor cells were detected by the markers, corresponding to a maximum of 24 and 22 % of undetected malignant cells, respectively.

### Comment

Here, we identified several proteins that could potentially serve as a suitable target for detecting breast cancer cells within ovarian autografts. One clear application for these markers is the use of NIRF imaging, a technique that can differentiate malignant tissues from non-malignant tissues without reducing the tissue’s viability [10, 33–35]. Designing a NIRF probe directed against E-cadherin shows particular promise, as E-cadherin was expressed by the majority (94 %) of invasive breast tumor cells and was absent on the surface of normal ovarian cells. However, a combination of tumor-selective probes will likely be needed to detect all tumor cells. Based on our results, a combination of probes against E-cadherin, EMA and Her2/neu seems suitable.

Metastatic spread requires the local invasion of the surrounding host tissue by cells that originated from the primary tumor, followed by intravasation in blood and lymphatic vessels, ultimately leading to the dissemination of tumor cells [36]. E-cadherin and EpCAM mediate cell–cell adhesion, and the downregulation or loss-of-function of these proteins enables cells to escape from solid tumors [19]. E-cadherin and/or EpCAM are not necessarily expressed in all tumor cells; therefore, metastatic tumor cells might not be detected in some tissues. Furthermore, the majority of metastatic lobular breast cancer cells, which lack E-cadherin expression, will not be detected using a specific anti-E-cadherin probe, even though lobular breast cancer cells are more likely to invade ovarian tissue compared to cells derived from ductal carcinomas [37, 38].

As mentioned above, we considered tumor cell membranes positive if they showed immunoreactivity of any intensity. As a result, some tumor cells might be more positive than others for the investigated markers. Yet, for NIRF imaging, the staining intensity is less important as long as a significant tumor-to-background-ratio can be achieved.

None of the premenopausal ovaries in our cohort had positive staining in either the stromal cells or the ovarian surface epithelium. However, all markers (except CEA and uPAR) were expressed on epithelial cells in inclusion cysts. Consequently, conjugating antibodies against these markers to a NIR fluorophore will illuminate invasive breast cancer cells in the ovary, as well as inclusion cysts. Because inclusion cysts might differ from metastatic breast cancer cells with respect to their fluorescent configuration, it might be possible to distinguish between these structures. The same strategy might be used to distinguish granulosa cells in primary follicles from metastatic breast cancer cells that express E-cadherin. In addition, full-field optical coherence tomography (FF-OCT), a non-invasive imaging technique that mimics conventional histopathology, might be very useful. In the field of dermatological oncology, FF-OCT has already been proven capable of visualizing sebaceous glands and adipose tissue surrounding hair follicles as well as small malignant skin tumors [39]. On high magnification, fine architectural details on the subcellular level can be recognized. Therefore, it is expected that FF-OCT will also be able to distinguish inclusion cysts from metastatic tumor cells in ovarian tissue.

One strength of our study is that we examined the expression of tumor markers in tumor tissues obtained from young breast cancer patients who met the criteria for ovarian tissue cryopreservation. Moreover, only biologically active ovaries were analyzed, giving the results clinical relevance, as ovarian tissue is generally cryopreserved before ovarian failure has occurred.

On the other hand, this study has some limitations that merit discussion. First, a relatively small sample size was examined. However, normal ovarian tissue from premenopausal patients is not readily available. We excluded ovaries that were removed due to the presence of a *BRCA* gene mutation, as such samples could contain primary ovarian tumor cells [40], that express the markers investigated in this study. To be certain, we also excluded ovaries from breast cancer patients with unknown mutation status. At the LUMC, normal ovarian tissue from premenopausal breast cancer patients was exclusively available from *BRCA* mutation carriers or women with unknown mutation status. As a consequence, ovaries from breast cancer patients were not included in this study. Malignant cells may also be present in ovaries that were removed due to endometrial carcinoma, uterine sarcoma, cervical squamous cell carcinoma, or contralateral ovarian carcinoma; however, the risk of false-positive results was relatively low in our cohort, as all primary tumors were diagnosed at an early stage and the lymph nodes in these patients were clear. Second, the expression of the markers was evaluated on primary invasive breast tumors, since a substantial cohort consisting of ovarian tissues containing breast cancer metastases is scarce. Finally, we examined the expression of tumor markers in relatively small tumor cores using a TMA approach. Yet, the TMA technique is considered an accurate method for examining protein expression in breast cancer tissues [41].

Recently, the intraoperative use of tumor-targeted fluorescent imaging yielded a high tumor identification rate and enabled the surgeon to detect metastases that could not be detected by visual observation [42]. For our purpose, tumor-specific NIRF probes could be administered intravenously prior to oophorectomy, after which the removed ovary is dissected into cortical ovarian strips. Detailed fluorescent images could then be obtained using multiphoton microscopy, which provides an inherent submicron spatial resolution that allows revelation of subcellular details with reduced phototoxicity and photobleaching [43, 44]. Because the NIRF signal lies beyond the red end of the visible spectrum, the signal has enhanced tissue penetration, enabling the identification of fluorescently labeled tumor cells that are located deep within the tissue. Moreover, the low autofluorescence of the tissue at the emission wavelength of the probe provides a high tumor-to-background ratio [45]. Because of these features and the fact that cortical ovarian fragments can be imaged from both the upper and lower side, thereby increasing the imaging depth even further, NIRF imaging is a promising technique for detecting tumor cells in cortical ovarian strips up to 2 mm in thickness.

In conclusion, we report the identification of tumor markers that may serve as a target for detecting breast cancer cells in ovarian tissue using robust imaging techniques such as NIRF imaging. Based on our analysis, E-cadherin is likely the most suitable target for designing a tumor-specific probe. Further research will focus on examining the expression of these markers on breast cancer metastases in ovaries, refining methods to distinguish breast cancer cells from ovarian inclusion cysts, and examining the clinical feasibility of applying NIRF imaging to the field of fertility preservation.
